# The gut–lung axis: a new perspective on the impact of atmospheric particulate matter exposure on chronic obstructive pulmonary disease

**DOI:** 10.3389/fimmu.2025.1657675

**Published:** 2025-11-04

**Authors:** Xichen Pang, Ping Huang, Sha Huang, Xiaoju Liu

**Affiliations:** ^1^ The First School of Clinical Medicine, Lanzhou University, Lanzhou, China; ^2^ Department of Gerontal Respiratory Medicine, The First Hospital of Lanzhou University, Lanzhou, China

**Keywords:** particulate matter, chronic obstructive pulmonary disease, immune, microbiota, oxidative stress

## Abstract

Environmental pollution is a serious public health problem closely related to various chronic respiratory diseases, such as chronic obstructive pulmonary disease (COPD), bronchial asthma, and lung malignancies. Atmospheric particulate matter (PM) is an important component of environmental pollution, and its influence on COPD has been shown to be related to inflammation, oxidative stress, immune imbalance, abnormal cell death, and cell aging. A growing body of evidence has shown that an imbalance of the lung and intestinal microbiota, as well as changes in metabolites, is closely related to the occurrence and development of PM-induced COPD. PM exposure damages the respiratory system and alters the structure and activity of the gut microbiome. The metabolites produced by the gut microbiome, in turn, disrupt airway immunity and exacerbate respiratory inflammation. Therefore, the bidirectional influence of PM on the gut–lung axis has attracted widespread attention. This review explores the mechanisms by which PM causes oxidative stress damage to the lungs and intestines, as well as the characteristics of the resultant immune imbalance and changes in the microbiota and metabolite products. It also describes how PM disrupts barrier function through microecological imbalance and how it participates in the progression of COPD via the gut–lung axis. These mechanisms highlight the potential of targeting the microbial flora as a new approach for treating COPD caused by environmental pollution.

## 1 Introduction

Chronic obstructive pulmonary disease (COPD) is a respiratory condition characterized by chronic respiratory symptoms and persistent airflow limitation, which are primarily triggered by toxic particles and gases from tobacco smoke and air pollution. It is a preventable and treatable disease of the respiratory system (1). The global burden of COPD is increasing as a result of population aging and environmental factors, imposing a heavy economic burden on society (1–3). Atmospheric particulate matter (PM) exposure leads to increased oxidative stress in the lungs, yet this effect remains localized to the lungs (4). However, patients with COPD often have multiple systemic manifestations, such as gastrointestinal disorders, osteoporosis, anxiety, and depression (1), suggesting that COPD is not merely an isolated pulmonary disease but a complex systemic inflammatory disease. Studying the potential pathogenic mechanisms of COPD is crucial for slowing the progression of this disease and reducing mortality.

The structural and functional stability of respiratory microorganisms plays a major role in the pulmonary defense mechanism of the host. Microbes and their metabolites are involved in regulating the mucosal immunity of the respiratory system, maintaining the integrity of the respiratory epithelial barrier, and resisting the invasion of pathogenic bacteria (5). Evidence indicates that COPD patients exhibit dysbiosis in their respiratory microbiota (6, 7), manifesting as reduced microbial diversity, decreased colonization of bacteria, and replacement of some commensal bacteria by pathogenic bacteria such as *Pseudomonas* and *Streptococcus* (7–9). Patients with COPD also exhibit gut microbial dysbiosis, characterized by reduced richness and diversity, a depletion of beneficial metabolite-producing bacteria, and an enrichment of those producing harmful metabolites (10, 11). The gut–lung axis is characterized by close cross-communication between the gut and the lungs. This communication mainly relies on immune mediators and metabolic pathways, among which the gut microbiome and its metabolites play a central role. In the healthy state, the gut microbiota maintains the integrity of the intestinal barrier by producing beneficial metabolites (such as short-chain fatty acids [SCFAs]) and systematically regulates immune homeostasis, thereby providing protection to the distal lungs (12). Disorders of the intestinal microbiota in COPD may lead to impaired barrier function and the entry of inflammatory metabolites related to the intestine (such as lipopolysaccharide [LPS]) into the bloodstream, triggering systemic low-level inflammation and stimulating the pulmonary immune response (11). Conversely, co-infection with the influenza virus and *Haemophilus influenzae* leads to more severe infiltration of inflammatory mediators in the lung tissue of COPD mice, and the imbalance of the intestinal microbiota is also associated with the levels of inflammatory cytokines in the lungs. This suggests that changes in the intestinal flora are related to inflammatory infiltration in the lungs (13).

The harm of environmental pollution to human health has aroused widespread concern. PM in the environment consists of complex compounds and is an important environmental risk factor for COPD. It mainly comes from industrial, agricultural production, human life, and production, such as tobacco, automobile exhaust, tire wear, biofuel combustion, coal, decoration materials, and plastic bottles. PM exposure contributes to the incidence of COPD and elevates the risks of outpatient visits, acute exacerbation, hospitalization, and mortality rates in COPD patients (14–17). Emerging evidence indicates that PM exposure not only alters the structure and composition of the respiratory microbiota but also disrupts multiple pulmonary metabolic pathways (18–20). PM can also be directly transported to the intestine through swallowing, thereby disrupting the ecological balance of the intestinal flora (21). The gut–lung axis may constitute the core mechanism by which PM accelerates the progression of COPD. Our review explores the effects of PM on the lungs and the intestines, and explores the core mechanism of the gut–lung axis in the development and progression of COPD, with the aim of identifying new treatment directions to counter the influence of air pollutants on COPD.

## 2 Effects of PM on lungs

### 2.1 Mucosal barrier damage and oxidative stress

The muco-ciliary barrier system is the first physical barrier of the respiratory tract against pathogens, mainly including ciliary mucus clearance, epithelial barrier function, and the secretion of some proteins and peptides with antimicrobial activity. An *in vitro* simulation experiment found that diesel exhaust particles (DEPs) decreased the expression of cilia-related genes in human bronchial epithelial cells and epithelial tight junction protein, increased mucus secretion, resulting in the epithelial mucosal barrier function (22). PM triggers pulmonary oxidative stress by generating excess reactive oxygen species (ROS) through the activation of oxidases, metabolic enzymes, and mitochondrial impairment. This overwhelms the antioxidant defense system, evidenced by suppressed nuclear factor erythroid 2-related factor 2 (Nrf2), superoxide dismutase (SOD), catalase (CAT), and glutathione (GSH) expression. Culminating in oxidative/antioxidant imbalance, protein carbonylation, lipid peroxidation, and DNA damage (23, 24).

### 2.2 The macro impact of PM on immune cells

**Figure 1:**
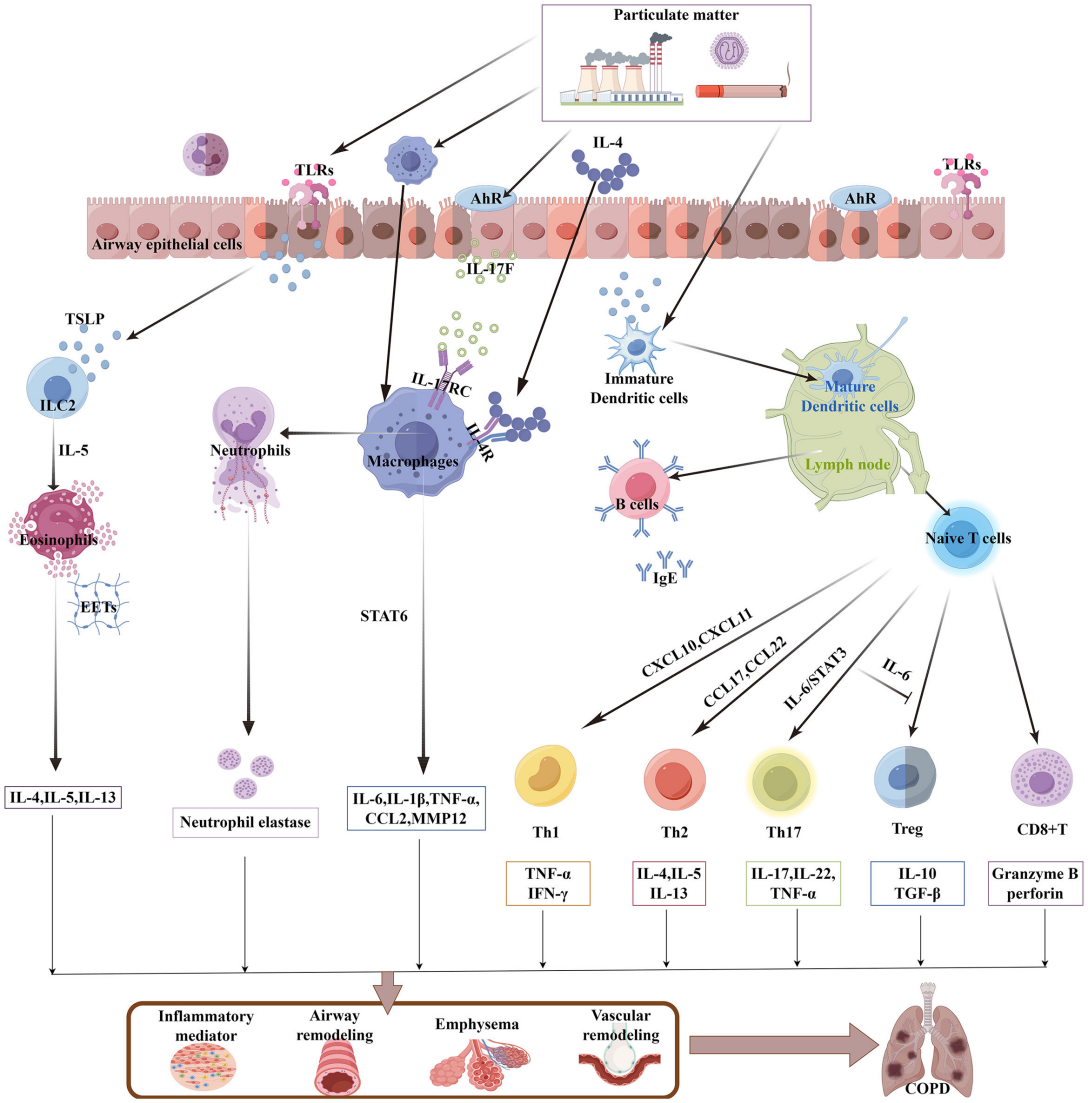
PM activates the lung immune system to participate in COPD. PM induces TSLP release from airway epithelial cells or directly activates DCs and alveolar macrophages, which in turn trigger additional innate immune responses (including eosinophils, neutrophils, and ILC2s). The activated DCs then migrate to lymph nodes, where they initiate adaptive immune responses. Naive lymphocytes are then activated and differentiate into T-cell subsets and mature B cells. A large number of proinflammatory cytokines, chemokines, and elastases contribute to the pathological development of COPD. PM, particulate matter; TSLP, thymic stromal lymphopoietin; DC, dendritic cell; ILC2, group 2 innate lymphoid cell; AhR, aryl hydrocarbon receptor; EETs, eosinophil extracellular traps.

PM enters the respiratory tract with breathing. Depending on the size of the PM, PM can deposit in any area from the bronchi to the alveoli, which leads to infiltration of plenty of immune cells in the lung tissue (25, 26). PM can activate the immune system, leading to airway inflammation through the release of immune mediators (**Figure 1**). This chronic immune-inflammatory stimulation can cause alveoli to rupture, resulting in emphysema, thickening of the airway walls, and narrowing of the lumen, all of which jointly cause obstructive ventilatory dysfunction. Studies have shown that an increase in various immune cells in the airways of patients with COPD, such as macrophages, neutrophils, T lymphocytes, and B lymphocytes (27). PM stimulated the infiltration of neutrophils, macrophages, eosinophils, and lymphocytes in the bronchoalveolar lavage fluid (BALF) and airway submucosa of mice (28). These abnormal immune cell infiltrations are closely associated with the airway remodeling and chronic inflammation in COPD.

### 2.3 PM on the microlevel of immune cells

Respiratory epithelial cells not only serve as the first line of defense for the airways but also possess various immune functions. The organic compounds and heavy metals in PM trigger cellular sensing mechanisms, such as interleukin (IL)-17A/IKKα and ROS/NF-kB through toll-like receptors (TLRs) and aryl hydrocarbon receptor (AhR), effectively stimulating the secretion of pleiotropic pro-inflammatory factor thymic stromal lymphopoietin (TSLP) by airway epithelial cells, to activate the innate immune response and the adaptive immune response successively (29, 30). In patients with COPD, TSLP can stimulate type 2 innate lymphoid cells (ILC2) to exert immune functions by producing IL-5, and promote the eosinophilic inflammatory response (31). Dendritic cells (DCs) are the most potent antigen-presenting cells and the key immune cells that connect innate immunity and adaptive immunity. PM can increase the number of DCs in the airways of patients with COPD (32). TSLP is also an activator for DCs (33), With the participation of DC, TSLP also recruits Th1 and Th2 cells through Th1 chemokines (CXCL10 and CXCL11) and Th2 chemokines (CCL17 and CCL22) (34), DCs can also be directly activated by PM, PM promotes the expression of CD80/major histocompatibility complex class 1 (MHC-I)/CD40 on bone marrow-derived DCs (BM-DCs) through multiple signaling pathways such as GATA3/MHC-R and TLR4-MYD88, homing to the lymph nodes and promote the development of lymphoid follicles, stimulate the initial lymphocytes to differentiate into T-cell subsets and mature B lymphocytes (35), they form a chronic inflammatory infiltration zone around the lung tissue and airways, which is known as “lymphoid follicles” (27), they are associated with airway remodeling. Furthermore, PM stimulates TLR2 on DCs, through the DNMT3a/c-Jun/AIF1 signaling pathway, induces the differentiation of Th17 cells (36, 37), further research has found that IL-6 can also reduce the inhibitory ability of regulatory T cells in suppressing T-cell proliferation (38), under their combined effect, it leads to the Th17/regulatory T-cell (Treg) imbalance in COPD.

Alveolar macrophages (AMs) are the core cells of the innate immune system. They have the functions of phagocytosing pathogenic bacteria and toxic particles, secreting cytokines, and chemotaxis (39). AMs can be activated by respiratory epithelial cells. Biomass smoke exposure stimulates the release of IL-17F by mouse lung epithelial cells, through paracrine mechanisms, which binds to the IL-17RC receptors on AMs, promoting the secretion of inflammatory factors such as IL-6 and CCL2 (40). AMs can engulf PM in diesel exhaust, as carbon particles can be observed deposited within the AMs (41). PM can directly act on AMs to exert different effects. Since the polarization of AMs plays a significant role in the progression of COPD, fine particulate matter, with aerodynamic diameter ≤ 2.5 µm (PM_2.5_) stimulated the high expression of IL-4 in mouse BALF, which a key inducer of M2 polarization, acting on the IL-4R on the surface of AM cells, inducing downstream STAT6 phosphorylation and nuclear translocation, binding to the Matrix Metalloproteinase -12 (MMP12) promoter region and promoting the expression of MMP12, causing excessive degradation of extracellular matrix (ECM), which is related to the destruction of lung tissue in COPD (42). Under continuous stimulation, AM shows decreased movement and phagocytic functions, unable to effectively remove apoptotic cells and inflammatory mediators. Instead, it continuously releases pro-inflammatory factors, maintaining the inflammatory state of COPD (39). In mice with COPD exposed to PM_2.5_, PM_2.5_ reduced NAD^+^/SIRT1 activity to increase the activity of histone acetyltransferases, resulting in elevated expression of pro-inflammatory genes in AM (43), releasing potent proinflammatory factors such as tumor necrosis factor (TNF)-α, IL-1β, and IL-8, causing a large influx of neutrophils from the circulation into the lung tissue (44). This is particularly evident during the acute exacerbation phase of COPD, as the persistent purulent sputum in the airways is an intuitive manifestation of excessive infiltration of neutrophils (45). Moreover, when neutrophils are clearing foreign substances, they release neutrophil elastase, which damages the elastic fibers of the alveoli and leads to emphysema (46).

CD8^+^ T cells are an important subgroup of T cells and are associated with tissue damage and the expansion of inflammatory responses. PM induces the aggregation of CD8^+^ T cells in the subepithelial layer of the airway and the alveolar septum of rats with COPD, to releases granzyme B and perforin, leading to an increase in cytotoxic T lymphocyte (CTL) response and resulting in lung tissue damage (47), It may be related to the fact that PM stimulates BM-DCs to highly express CD83/CCR7, thereby promoting the proliferation of CD8^+^ T cells (48). However, activated lymphocytes or inflammatory mediators reach the intestine, exposing the intestine to continuous inflammatory signals. Thus, the immune disorder in the lungs caused by PM exposure is the core of the vicious cycle of the gut–lung axis.

### 2.4 Changes in lung microorganisms and metabolites

Microorganisms have been confirmed to exist in the lungs, and their colonization occurs during the early stages of life, such as at birth, during the neonatal period, and even during the fetal stage (49, 50). Colonization of lung microorganisms in the early stages of life plays a crucial role in orchestrating alveolar development and pulmonary immune maturation (51). The lung microbiota plays multiple roles in the lung ecosystem, including the metabolism of nutrients, immune regulation, and maintenance of lung homeostasis. The composition of the lung microbiota and the levels of metabolites are influenced by various factors such as the environment, disease status, diet, and immune conditions (5).

PM affects the balance of lung microecology and alters the composition and metabolites of lung microorganisms. Both clinical and animal studies have demonstrated that exposure to PM_2.5_ alters the composition of oropharyngeal microflora. Specifically, it increases the overall bacterial load and diversity while reducing the population of beneficial bacteria like *Lactobacillus*. Conversely, PM_2.5_ promotes the growth of pathogenic bacteria, including *Streptococcus* and *Staphylococcus*. These shifts collectively impair the defensive function of the upper respiratory tract and elevate the risk of infection (52, 53). Epidemiological studies have revealed that PM alters airway microecology and affects lung function, and that forced expiratory volume in one second (FEV1) is significantly correlated with airway microbiota characteristics (20). At the phylum level, Actinobacteria, Proteobacteria, Firmicutes, and Bacteroidetes are the major lung flora in healthy mice (54, 55); the pathogenic bacteria, inorganic salts, and organic compounds carried by PM are critical factors affecting the lung microenvironment and structure of the microbial flora (20, 56), and their effect is persistent (20). Moreover, the effects of PM on pulmonary microorganisms vary with exposure time, concentration, exposure mode, and regional differences in respiratory tract flora (57, 58).

The PM-induced changes in the lung microbiome are closely related to the occurrence of COPD and vary with the duration of PM exposure. In the early stages of exposure to moderate-to-high levels of PM_2.5_, the abundance and diversity of microorganisms in the airways increase, and the major flora in the lungs are Proteobacteria, Bacteroidetes, Cyanobacteria, and Firmicutes (18). Subchronic exposure to PM_2.5_ decreases the abundance of Firmicutes, Bacteroidetes, Actinobacteria, Chloroflexi, and Euryarchaeota in BALF at the phylum level, while the abundance of Proteobacteria, Tenericutes, Planctomycetes, and Acidobacteria increases (19). Epidemiological studies have shown that various respiratory diseases are characterized by a decrease in the abundance of Bacteroides and an increase in the abundance of Proteobacteria (59). Mechanistic insights from animal models reveal that the abundance of Bacteroidota was negatively correlated with the levels of IL-1β, TNF-α, and malonaldehyde (MDA) and positively correlated with the levels of CAT and GSH; these findings suggest that a decreased level of Bacteroidota is involved in the imbalance of inflammatory response and oxidative stress (60). This imbalance causes the pulmonary system to remain in a low-level but continuously activated inflammatory state, which is associated with airflow limitation and lung tissue damage in COPD (60). PM_2.5_ exposure can increase the abundance of Proteobacteria (60), and other animal studies have shown that exposure to PM_2.5_ in real environments increased the abundance of Alphaproteobacteria, Pseudomonadaceae, and Desulfovibrionaceae belonging to the phylum Proteobacteria; more importantly, the inflammatory response of lung tissue has been shown to be related to excessive proliferation of Proteobacteria (54). In patients with COPD, the number of Proteobacteria also increased and was higher during the acute exacerbation period than during the stable period (61). The increase in the abundance of Proteobacteria may be due to the fact that PM_2.5_ stimulates the secretion of Muc5ac in the airway, which Proteobacteria can metabolize. PM_2.5_ also promotes AMs to produce peroxynitrite, which provides nutrients for anaerobic respiration and selective growth of Proteobacteria. In addition, Proteobacteria are rich in lipopolysaccharides and flagellin, which are recognized by TLRs of AMs to cause oxidative stress damage to cells and release inflammatory mediators such as IL-1β, IL-6, TNF-α, and nuclear factor (NF)-κB, thereby recruiting more neutrophils and macrophages. Therefore, the increased abundance of Proteobacteria is closely related to respiratory tract pathogen infections, pulmonary oxidative stress, and inflammatory responses (60, 62, 63), which are closely associated with COPD. In comparison with healthy individuals, patients with COPD show significant differences in the airway microbiome, which is characterized by reduced microbial diversity, and the more severe the airflow limitation, the less diverse the microbial species in the sputum (64). An increase in pathogenic bacteria such as *Streptococcus* and *Moraxella* can release pathogen-associated molecular patterns (PAMPs), which, in turn, trigger acute exacerbation of COPD (8).

In addition, high concentrations of PM_2.5_ in the ambient air can accelerate the progression of COPD by increasing the abundance and variety of bacteria in the BALF of patients with stable COPD (65). The imbalance in the lung microbiota is significantly correlated with decreased lung function, as well as with the inflammatory factors IL-1β, IL-10, IL-17A, and interferon (IFN)-γ (66). Research has found that changes in the lung microbiota have been associated with the season of PM_2.5_ exposure and the severity of COPD. When patients with COPD exposed to PM_2.5_ were followed up by collecting their sputum samples every 3 months according to the season, the abundance in the lung microbiota was the highest in summer, with increased α-diversity and reduced abundance of Proteobacteria, Firmicutes, and Bacteroidetes. The influence of PM_2.5_ on the respiratory microbiome was more obvious in patients with moderate COPD and less pronounced in those with severe COPD (58). Notably, due to individual differences in respiratory microorganisms, microbial composition varies in different parts of the respiratory tract, and differences have been observed in individual immune statuses and susceptibility. Moreover, the composition of PM is also affected by climate, region, and source. These reasons can lead to different respiratory microbial changes under PM exposure in different studies. In addition, at the phylum level, the same bacterial phyla contain both probiotics and pathogenic bacteria; therefore, with regard to the changes in the main microorganisms in the lung, some studies showed an increase in the abundance of flora, while others showed the opposite results for phyla such as Firmicutes.

PM exposure alters the levels of pulmonary metabolites involved in metabolic pathways, disrupting various energy metabolism pathways, such as lipid, amino acid, and sugar metabolism. The molecules involved in these processes are key mediators of inflammatory signal transduction. Therefore, PM jointly promotes the pathological process of COPD through metabolic disorders of the lungs. Exposure to PM_2.5_ causes alterations in metabolites such as fumaric acid and the branched-chain amino acids valine and isoleucine, choline, creatine, betaine, citrate, pyruvate, and glutamine in mouse BALF, which are strongly associated with several typical lung microbiota. These metabolites are involved in various metabolic pathways, including the tricarboxylic acid cycle as well as pyruvate, purine, pyrimidine, and choline metabolism. PM leads to a decrease in the levels of fumaric acid, an important intermediate metabolite in the tricarboxylic acid cycle, and also causes a reduction in the levels of valine and isoleucine, which are essential branched-chain amino acids involved in energy metabolism (18, 19). A decrease in valine and isoleucine may also affect the antioxidant defense system (67), making lung tissue more susceptible to oxidative damage. And that fumaric acid inhibits the MAPK-dependent NF-kB signaling pathway, reduces the expression of TNF-α and CCR3, and alleviates the inflammatory response (68). All of these factors are related to the progression of COPD. Meanwhile, PM_2.5_ stimulates the metabolism of linoleic acid and upregulates the metabolism of arachidonic acid downstream; arachidonic acid and its metabolites play key roles in many inflammatory diseases (55). Therefore, the disruption of multiple energy metabolism pathways by PM_2.5_ is one of the causes of chronic inflammation in the lungs and lung function impairment in patients with COPD.

A clinical study revealed that in patients with COPD exposed to PM, the lung metabolic flora also has active metabolic ability, especially for the transport and metabolism of amino acids, inorganic ions, and carbohydrates. These active metabolic functions cause increased nutrient consumption, resulting in decreased immune function and increased risk of infection. The findings also suggested that PM promotes COPD progression in part through the altered abundance of airway microbes that accelerate airway repair and airway remodeling processes (65). Phosphatidylcholine (PC) is the most abundant phospholipid in mammalian cell membranes and subcellular organelles, and it plays an important role in maintaining cell membrane stability. In one animal study, PM_2.5_ was shown to damage alveolar type II cells of rats to reduce the secretion of the surfactant PC, which caused alveolar septa fracture and alveolar cavity fusion; these changes reduced the levels of the lung lipid metabolite lysophosphatidylcholine, which is associated with the prevention of airway inflammation (69). PM_2.5_ exposure also increased the accumulation of free fatty acids, acylcarnitines, lipid metabolites, and lipid toxicity associated with pathological damage (70). Impaired membrane stability promotes inflammatory factors and metabolites into the lymphatic and circulatory systems, and participates in the development of COPD through the gut–lung axis.

## 3 Effects of PM on the intestine

The harmful effects of PM on health extend beyond the respiratory system. Since both the lungs and intestines are closely connected to the external environment, PM may concurrently damage both organs (**Table 1**). PM-induced intestinal damage and dysfunction are caused by oxidative stress injury, immune imbalance, and microecological imbalances.

**Table 1:** PM causes both respiratory system and intestinal changes.

Species	PM intervention method	Sample sources	Changes in the respiratory system	Changes in the intestinal	References
Male Balb/c mice	Intratracheal instillation of PM_2.5_ for 10 weeks	Lung/ileum tissues	Pulmonary injury and inflammatory infiltration: (1) Alveolar septal thickening. (2) Infiltration of inflammatory cells. Changes in lung microbiome: (1) At the phylum level, Firmicutes and Bacteroidetes increased, while Proteobacteria decreased. (2) At the genus level, *Streptococcus*, *Flavobacterium*, *Propionibacterium*, and *Neisseria* increased, while *Halomonas* and *Lactobacillus* decreased. Changes in lung metabolite: (1) Hypoxanthine, guanosine, and hepoxilin B3 increased.	Changes in gut microbiome: (1) At the phylum level, Firmicutes and Proteobacteria increased, while Bacteroidetes and Bacteroidetes/Firmicutes decreased. (2) At the genus level, *Prevotella* increased, while *Oscillospira*, *Bacteroides*, *Alistipes*, *Helicobacter*, and *Lactobacillus* decreased. Changes in gut metabolites: (1) Spermidine, l-tryptophan, and serotonin increased.	(55)
Wild-type (WT) mice, Nrf2^−/−^ (KO) mice	Daily exposure for 16 h/day for 6/12 weeks	Lung tissue/cecal feces	Changes in lung microbiome: (1) At the phylum level, Proteobacteria increased, while Bacteroidetes and Firmicutes decreased. (2) At the class level, Alphaproteobacteria, Pseudomonadaceae, and Desulfovibrionaceae increased.	Changes in gut microbiome: (1) At the genus level, *Sutterella* increased.	(54)
C57BL/6 mice	Tracheal instillation of 12.5 mg/kg PM_2.5_ on days 8, 11, 14, and 17.	BALF/lung tissue/cecal content	Pulmonary injury and inflammatory infiltration: (1) Pneumoedema. (2) Thickened alveolar septum. (3) Increased levels of IL-1β, IL-6, and TNF-α in BALF. (4) Reduced SOD, and increased MDA and MPO. Changes in lung microbiome: (1) At the phylum level, Actinobacteriota decreased, while Firmicutes increased. (2) At the genus level, *Rhodococcus* and *Raoultella* decreased. (3) At the family level, Desulfovibrionaceae, Moraxellaceae, Sphingobacteriaceae, and Xanthomonadaceae increased.	Intestinal mucosal barrier damage: (1) Goblet cells in the colon and ileum decreased. Changes in gut microbiome: (1) The richness of intestinal microbial communities decreased. (2) At the phylum level, Desulfobacterota increased, while Bacteroidetes decreased. (3) At the genus level, *Mucispirillum* increased. (4) At the family level, Desulfovibrio and Deferribacteraceae increased. Changes in cecal metabolite: (1) Butyric acid decreased.	(127)
BALB/c mice	Exposed to concentrated ambient PM_2.5_	BALF/lung/fresh stool pellets	Pulmonary injury: (1) Thickening of the alveolar wall and pulmonary edema. (2) Bronchopneumonia and interstitial pneumonitis. Changes in lung microbiota: (1) At the phylum level, Firmicutes, Bacteroidetes, Actinobacteria, Chloroflexi, and Euryarchaeota decreased, while Proteobacteria, Tenericutes, Planctomycetes, and Acidobacteria increased. Changes in BALF metabolites: (1) Choline, TMAO, creatine, betaine, citrate, pyruvate, and glutamine decreased.	Intestinal damage: (1) Shortening of jejunal villi. Changes in gut microbiota: (2) Fecal bacterial richness decreased. (3) At the phylum level, Firmicutes, Actinobacteria, and Deferribacteres decreased, while Bacteroidetes and Cyanobacteria increased.	(19)
1.5-year-old Fischer 344 rats	Exposure to traffic-related pollutants for 3 months	Lung/fecal samples	Changes in lung microbiota: (1) At the phylum level, Firmicutes and Bacteroidetes increased, while Proteobacteria decreased.	Changes in gut microbiota: (1) At the phylum level, Bacteroidetes increased, while Firmicutes decreased.	(100)
Sprague–Dawley rats	Exposed to biomass fuel/motor vehicle exhaust throughout the body for 4, 12, and 24 weeks	BALF/lung tissue/colon contents	Pulmonary inflammation and injury: (1) Total leukocyte, alveolar macrophage, and neutrophil counts increased. (2) Bronchial wall thickening. (3) PEF, FEV20/FVC, and FEV100 decreased. Changes in lung microbiota: (1) Microbial changes varied depending on exposure time.	Changes in gut microbiota: (1) Richness of OTUs and alpha diversity decreased. (2) Microbial changes varied depending on exposure time. Changes in colon metabolites: (1) Total SCFAs and acetic acid decreased.	(11)
C57BL/6N mice	Exposed to PM_2.5_ via intratracheal instillation	Lung tissue/colonic contents	Pulmonary injury and inflammatory infiltration: (1) Alveolar wall thickening; (2) Neutrophil infiltration in the alveolar space; (3) Levels of IL-1β, IL-6, IL-17, and TNF-α increased in BALF. (4) Levels of MDA and ROS were elevated, while GSH and T-SOD decreased in BALF. Changes in lung metabolites: (1) Arachidonic acid metabolism and alpha-linolenic acid metabolism were altered.	Changes in gut microbiota: (1) α-Diversity decreased. (2) At the phylum level, the abundance of Firmicutes and Bacteroidota increased, while the abundance of Verrucomicrobiota, Proteobacteria, Actinobacteriota, and Acidobacteriota decreased.	(128)
C57BL/6J mice	Whole body exposure to aerosols of carbon black (CB), ozone (O_3_), or a mixture of CB + O_3_	Lungs/colon contents	Pulmonary injury and inflammatory infiltration: (1) Eosinophils, neutrophils, and B lymphocytes increased, while T cells decreased. Changes in lung microbiota: (1) At the phylum level, Proteobacteria decreased. (2) At the family level, Caulobacteraceae and Gammaproteobacteria incertae sedis increased, whereas Pleomorphomonadaceae and Moraxellaceae decreased.	Changes in gut microbiota: (1) At the phylum level, Bacteroidetes and Firmicutes increased. (2) At the family level, Lactobacillaceae and Ruminococcaceae increased, while Clostridiaceae decreased.	(129)

### 3.1 Oxidative stress and immune imbalance

Exposure to PM increases the risk of intestinal disease, such as inflammatory bowel disease and appendicitis (71). PM entering the intestine can directly or indirectly impair intestinal barrier function by destroying intestinal epithelial cells. Increasing animal studies have found that PM weakens intestinal barrier function by shortening jejunum and ileum villi and inhibiting intestinal epithelial mucus secretion in mice (19, 72). Subsequently, polycyclic aromatic hydrocarbons (PAHs), heavy metals, and trace elements in PM enter intestinal epithelial cells through passive transfer, diffusion, and active transport, causing intestinal epithelial injury through oxidative stress (72–74). PM_2.5_ induced excessive ROS production in mitochondria of intestinal epithelial cells, increased the lipid peroxide MDA, and decreased the antioxidant oxidase SODs, resulting in oxidation/antioxidant imbalance. Additionally, PM_2.5_ induced apoptosis of intestinal epithelial cells by lipid peroxidation and damaged the cytoskeleton microtubule structure (75, 76). Ultrafine PM upregulates the expression of lipid oxidation metabolites HETEs and HODEs in the intestine, causing an increase in the proinflammatory lipid PGD2 (72). PM_2.5_ induces oxidation-dependent nuclear NF-κB activation, which promotes the release of proinflammatory cytokines IL-6, IL-8, and TNF-α in the intestine; these factors contribute to intestinal inflammation (76, 77). In addition, PM inhibits the expression of the intercellular adhesion proteins E-cadherin and the tight junction protein ZO-1, resulting in increased intestinal permeability, allowing microbial metabolites and PM to enter the circulatory system through the damaged intestinal epithelial barrier (76, 78). These are regarded as the crucial pathological and physiological bases for the intestinal damage caused by PM exposure in COPD.

PM participates in inflammation by disrupting both the innate and adaptive immune systems of the intestine. Studies have found that PM causes a large number of macrophages and neutrophils to infiltrate the villi area of the intestine (72). PM causes an imbalance of the Th17/Treg ratio, in which the frequency of Th17 cells increases while the frequency of Treg cells decreases. PM also decreased the numbers of CD4^+^ T and B220^+^CD69^+^ cells in intestinal Peyer’s patches, reduced the secretion of the anti-inflammatory cytokine IL-10, and increased the proinflammatory cytokine TNF-α (79, 80). Moreover, innate immunity can interact with adaptive immunity; the regulatory dendritic cells (rDCs) in mesenteric lymph nodes can promote the differentiation of CD4^+^Foxp3^+^ T cells, exerting immune tolerance (81). These inflammatory cytokines and immune cells enter to mesenteric lymph nodes through the damaged intestinal barrier and reach the lungs by the gut–lung axis to damage lung tissue.

### 3.2 Effects of PM on intestinal microorganisms and metabolites

The intestinal microbiota consists of a complex microbial system that colonizes the intestinal tract. The balance of the intestinal microbiota is crucial for maintaining human health, enabling the intestines to perform their normal physical barrier function, shape a normal immune system, and promote the absorption of nutrients. The intestinal microbiota is also influenced by various factors, such as the environment, diet, and health status. PM in the oropharynx enters the gastrointestinal tract through swallowing and disturbs the intestinal microecology, manifesting as changes in the composition and diversity of the intestinal flora. The inorganic salts and organic matter in PM alter the intestinal microenvironment and influence the colonization of microorganisms in the intestine. PM that enters the intestine is metabolized into toxic substances by specific microbiota, which directly affect the survival of microorganisms and damage epithelial function (82). The impact of PM on the gastrointestinal microbiota extends throughout the gastrointestinal tract, with changes gradually becoming more pronounced from proximal to distal regions, and the most significant differences are observed in the fecal microbiota (83). This is because the bioaccessibility of PM is influenced by the particle size, the composition of the digestive fluid—including its pH—the levels of the digestive enzymes pepsin and trypsin, and the chemical properties of metal ions (74). Emerging evidence has shown that the intestinal microbial flora in patients with COPD is imbalanced (84, 85). Exposure to PM leads to an imbalance in the intestinal flora, which is associated with the onset of COPD. In a rat model of COPD exposed to environmental PM, a decrease in microbial richness and diversity was observed. This occurred concurrently with PM-induced pulmonary emphysema and the infiltration of macrophages and neutrophils, suggesting a possible relationship between the gut microbiota and COPD (11). An animal experiment employing fecal microbiota transplantation (FMT) confirmed that the gut microbiota affects pulmonary immune status and lung function in COPD. FMT alleviated emphysema in mice with COPD exposed to cigarette smoke (CS) and improved lung function and systemic inflammatory responses (10). Studies have also shown a reduction in the diversity of intestinal bacteria in coal miners, as well as a correlation between the composition of intestinal microorganisms and lung function impairment (86). These findings indicate that disorders of the intestinal flora are closely associated with pulmonary inflammation and decreased lung function in COPD.

PM damages the intestinal barrier by altering the intestinal flora. The intestinal barrier serves as the first line of defense against harmful substances entering the body from the intestine. The intestinal microbiota, as a complex ecosystem, not only constitutes a part of this defense system but also plays an important role as a regulator and maintainer of the system. In the early stages of PM exposure, dysbiosis of the intestinal microbiota first causes damage to the intestinal mucosal barrier (73). DEPs reduce the thickness of the acidic and neutral mucus layers in the colon, and mucus loss occurs before inflammatory infiltration (73). Another animal study found that short-term PM exposure did not cause colon damage or inflammation, while the intestinal microbial diversity was reduced and the total bacterial load was increased. An increase in *Verruca* microbacteria and *Proteobacteria* promoted the degradation of intestinal mucus, resulting in weakening of the intestinal mucus barrier. PM_2.5_ also inhibited the production of butyrate by decreasing the abundance of *Lactobacillus*, as butyrate is the main energy source for intestinal epithelial cells. These results indicate that PM_2.5_ exposure may begin to weaken the intestinal barrier by disrupting the intestinal microflora, which subsequently leads to intestinal villi destruction and an intestinal inflammatory response (87). Short-term PM exposure caused a brief increase in the abundance of gut *Lactobacillus*, which may represent a self-protective mechanism in the host (73). Under continuous exposure to concentrated ambient PM_2.5_ (CPM), the intestinal mucus of mice became thinner, the villi of the ileum became shorter, the tight junctions of the intestinal epithelium were damaged, and the abundance of *Eubacterium rectale* and *Lactobacillus*—both believed to have protective effects on the intestinal barrier—was altered (88). These findings provide a theoretical basis for the reverse entry of intestinal inflammatory mediators and the proinflammatory metabolite LPS into the lungs (11).

PM causes intestinal inflammation by disrupting the gut microbiota. Exposure to PM leads to a decrease in the abundance of *Agathobacter* and *Romboutsia*, which exhibit anti-inflammatory activity in the intestines, and an increase in the abundance of proinflammatory bacteria such as *Klebsiella*, *Clostridium*, *Escherichia*, and *Haemophilus* (86, 87). *Klebsiella* and *Haemophilus* are common pathogens in COPD, contributing to frequent acute exacerbations of the disease and accelerating the deterioration of lung function (64, 89). Thus, disruption of the intestinal flora can cause intestinal inflammation, which spreads to the lungs. Long-term exposure to PM increases the abundance of *Akkermansia* within the Bacteroidetes phylum, which can trigger intestinal inflammation. The levels of IL-6 and IL-10 were positively related to Parabacteroides, while the levels of IL-17A and IL-17F were positively correlated with *Prevotella*, which is believed to be associated with lung inflammation (90). These findings indicate that the PM-induced changes in intestinal microbiota and the resulting intestinal inflammation infiltration contribute to the reverse transfer of inflammation and the development of COPD.

PM disturbs the intestinal flora and various metabolites. The PM-induced imbalance in the intestinal flora leads to a decrease in the production of beneficial metabolites, such as SCFAs (91), and an increase in harmful metabolites, such as LPS (11). Patients with COPD also show decreased diversity of intestinal flora; for every 10-μg/m^3^ increase in PM_2.5_ concentration, the α-diversity of the intestinal flora decreases by 2.16%, the relative abundance of Bacteroidetes declines, and the fecal levels of beneficial SCFAs—including isobutyric acid and isovaleric acid levels—decrease with worsening disease (85, 92). Similar to the results of clinical studies, exposure to ambient PM for 2 months significantly reduced the abundance of intestinal microbiota in mice, reducing the abundance of Firmicutes and *Actinomyces* and increasing the abundance of Bacteroides and Cyanobacteria, which have low basal abundance (19). With prolonged exposure to PM, the diversity and abundance of intestinal flora in rats decreased. PM exposure also reduced the level of SCFAs in the colon contents; SCFAs function as a substrate for energy metabolism, improve intestinal barrier function, and reduce intestinal bacterial translocation. SCFAs also activate the Nrf2 signaling pathway to maintain the balance between oxidation/antioxidation, reducing the oxidative damage of PM to cells (11, 93). This may be related to the decreased abundance of several SCFA-producing bacteria, including *Lactobacillus*, in the gut (87). PM disrupts multiple metabolic pathways by altering the homeostasis of the bacterial community. The abundances of *Enterococcus faecium* and *Bacteroides fragilis*, which are involved in glycolysis, in the intestinal tract are closely associated with air pollutants. The NO_2_ level in air pollutants is negatively correlated with the serum levels of the metabolite melatonin, while the CO level is negatively correlated with the serum level of the metabolite C-8C1P, which is closely related with disturbances in lipid and fatty acid metabolism in patients with Acute Exacerbation of COPD (AECOPD) and plays a protective role in the inflammatory response (92). In an animal study, the diversity and abundance of the intestinal flora species decreased in Sprague–Dawley (SD) rats administered oral gavage of PM_2.5_, confirming that PM_2.5_ perturbs various metabolic pathways in serum by altering the composition of intestinal microorganisms. The most disturbed metabolic pathways are glycerophospholipid metabolism, which affects the ability to maintain cell membrane stability, transport triglycerides, and participate in inflammation, and linoleic acid metabolism, which is related to oxidative stress (94). Microbial functional analysis based on Kyoto Encyclopedia of Genes and Genomes (KEGG) enrichment showed that CPM-exposed mice had increased susceptibility to infectious diseases and viral infections. Moreover, basic metabolic processes, such as amino acid metabolism, lipid metabolism, and carbohydrate metabolism, were significantly attenuated in these mice (88). In addition, the intestinal microecological imbalance caused by PM varies depending on exposure time, dose, composition, and source (11, 95).

## 4 PM and the gut–lung axis in COPD

**Figure 2:**
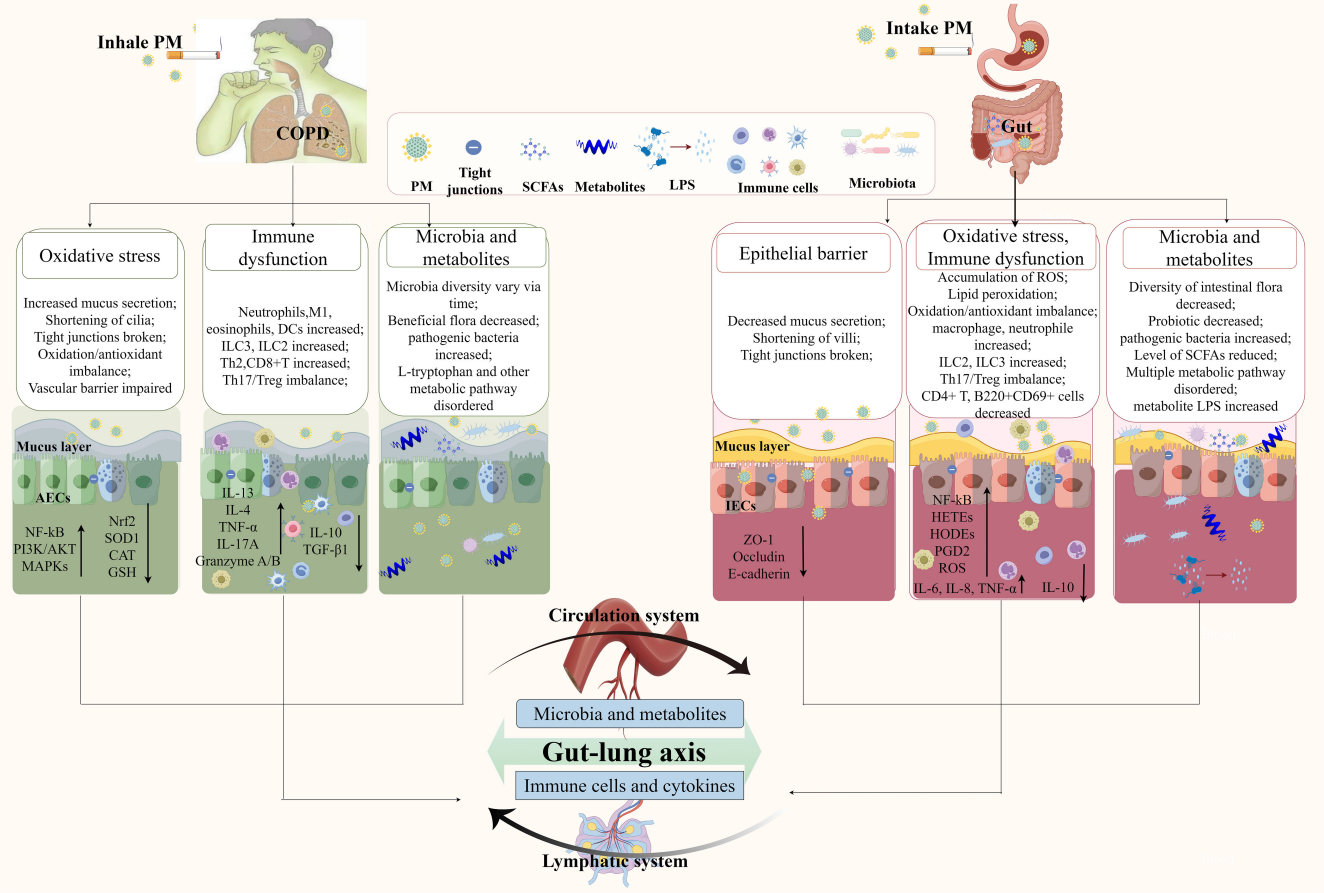
PM affects the gut–lung axis and is involved in COPD. Through the respiratory tract pathway, inhaled PM disrupts the lung epithelial barrier, immune homeostasis, lung microbiota, and metabolites, leading to the release of inflammatory mediators and abnormal metabolites that enter the bloodstream, disrupt the intestinal flora, and trigger intestinal inflammation and dysfunction. Through the digestive tract pathway, PM that enters the intestine via swallowing weakens the intestinal mucosal barrier, activates intestinal mucosal immunity, and disrupts the intestinal microecological balance. These abnormal metabolites and immune mediators reflux into the lungs, ultimately exacerbating the progression of COPD. AECs, alveolar epithelial cells; IECs, intestinal epithelial cells; PM, particulate matter; SCFAs, short-chain fatty acids.

The close connection between the lungs and intestine plays a key role in the pathophysiology of PM-induced COPD. A large national clinical study showed that patients with COPD are more likely to develop intestinal disease, and the incidence increases as COPD worsens (96). Changes in the intestinal flora and metabolites can affect the progression of COPD. As shown in **Figure 2**, PM enters the body through two pathways and interacts with the gut and lungs of patients with COPD through the gut–lung axis. The joint participation of the circulatory and lymphatic systems creates a close network of remote interactions between the two major organs, the lungs and the intestines.

The effects of PM on the gut–lung axis are mediated by immune-inflammatory mechanisms. Immune cells distributed on the mucosal surfaces of the body can communicate with each other across different mucosal tissues. Inflammatory cytokines are the common molecular basis for mucosal immunity in different parts and also serve as important immune mediators in the gut–lung axis. Activation of pulmonary immunity also increases the sensitivity of intestinal inflammation to environmental stimuli. An animal study showed that exposure to CS increased the release of IL-17A by Th17 cells in the lungs, and IL-17A increased the number of neutrophils in the circulation, activating the systemic inflammatory response. Neutrophil recruitment to the intestinal tissue is involved in intestinal pathological damage and is accompanied by increased secretion of IL-6 and IL-17A (97). A study using IL-17A gene knockout and anti-Ly6G depletion of neutrophils confirmed that the IL-17A–neutrophil axis was the indirect cause of the enhanced intestinal inflammatory response driven by CS exposure (97), illustrating the crucial role of pulmonary immune homeostasis in the tolerance of the lungs to environmental stimuli in the gut. The abnormal immune-inflammatory state in the lungs of patients with COPD also disrupts the intestinal microecological balance. Viral and bacterial infections are the main triggers of acute exacerbations of COPD. Infection with the influenza virus and *Haemophilus influenzae* leads to infiltration of neutrophils, natural killer cells, and macrophages in the lungs of COPD mice exposed to CS, as well as elevated levels of the inflammatory cytokines TNF-α, IL-1β, and IL-22. These inflammatory mediators enter the intestine through the circulatory system, causing a decrease in intestinal symbiotic bacteria and an increase in pathogenic bacteria, which is correlated with high levels of pulmonary inflammatory factors (13). Imbalance in the ratio of Firmicutes to Bacteroidetes in the intestinal microbiota can also trigger inflammatory signals (13), which may, in turn, exacerbate pulmonary inflammation. Furthermore, the activated intestinal immune system, as a remote inflammatory amplifier, actively releases proinflammatory signals that feed back to the lungs, exacerbating the pathological process of COPD. Under pathological conditions, ILC2s in the intestinal mucosa are activated and migrate from the intestine to the lungs through the lymphatic system and blood circulation. Inflammatory ILC2 (iILC2) entering the lung tissue then upregulate the expression of major histocompatibility complex class 2 (MHC-II) or secrete IL-13 and IL-4, providing a microenvironment for Th2 cell differentiation, promoting Th2-type immune responses, and transferring inflammation from the gut to the lungs, participating in the pathogenesis of COPD (98). The oral administration of traditional Chinese medicine formulas can maintain the Th17/Treg immune balance in the intestines of COPD mice exposed to PM, which can correct the Th17/Treg immune imbalance in the lungs, reduce inflammatory infiltration, and improve lung function (99). This bidirectional crosstalk involving immune-inflammatory mediators demonstrates the pathogenic mechanism of systemic inflammation in COPD caused by PM.

Furthermore, the interaction of the gut–lung axis is also realized through microbial translocation and changes in metabolites. Intestinal ecological disorders are closely correlated with changes in the composition of lung microbiota. A recent study showed that intratracheal instillation with PM_2.5_ changes the composition of the lung microbiota and triggers changes in lung metabolites, resulting in the upregulation of l-tryptophan metabolism, which is a key component and biomarker of the pulmonary cascade to the intestine; reduction of *Helicobact*er and *Spirillum* in the intestinal flora; and regulation of gastrointestinal motility and inflammation (55). PM causes proliferation of Bacteroidetes in both the lungs and intestines of mice (100), indicating possible microflora translocation. In mice, exposure to PM_2.5_ causes a decrease in Firmicutes and Actinomycetes in the lungs and intestines, and the high correlation between intestinal differential flora and serum metabolites and between pulmonary differential flora and BALF metabolites suggests that microbial flora and systemic metabolites may be involved in the pulmonary and intestinal interactions caused by PM_2.5_ (19). PM may directly disrupt the balance of the intestinal microbiota, and experimental studies on FMT have demonstrated that intestinal microbial imbalance plays a major role in the exacerbation of COPD. In a rat model of COPD exposed to PM, gut microbial diversity and abundance, as well as beneficial flora, decreased, although no significant changes were observed in lung microbes (11). The imbalance in the intestinal flora aggravates the deterioration of COPD. It might be the cause that PM disrupts the intestinal microbiota, activating immune cells in the intestine and causing them to migrate to the lungs, which are in an inflammatory state, and thereby exacerbating lung inflammation. An experiment employing intestinal microbiota transplantation confirmed this explanation. In this experiment, fecal flora isolated from COPD patients with GOLD stages I–II and III–IV disease were transplanted into mice that developed COPD after exposure to biomass smoke. The mice that received the intestinal microbiota of COPD patients showed worse lung function, which was accompanied by increased airway remodeling and greater mucus secretion, triggering higher expression of the inflammatory cytokines IL-1β and TNF-α in the serum, and increasing the number of neutrophils and T cells in the BALF (85).

The metabolic changes driven by the gut microbiota are important mediators of the gut–lung axis. PM disrupting the gut microbiota can also lead to abnormal purine metabolism (101), which results in the production of oxygen-free radicals, causing oxidative stress in the lungs. Moreover, purine metabolism disorders are associated with deterioration of lung function (102), which may explain the mechanism by which PM causes lung damage in COPD by disrupting gut microbiota. However, further interventional studies are needed to confirm this. Intestinal LPS is mainly released by Gram-negative gut bacteria, disrupting the intestinal epithelial barrier and activating inflammatory responses. PM induces the entry of LPS into the lung tissue through the circulatory system to mediate the inflammatory response and damage the lung tissue. Serum LPS levels are also significantly positively correlated with the mean linear intercept of the lung (72). The mechanism underlying this correlation may involve the leakage of LPS from intestinal microorganisms into the lungs, which then recognizes and activates the TLR4/NF-κB signaling pathway in lung epithelial cells to promote the inflammatory response in the lungs and participate in COPD (103). These findings indicate that imbalances in the intestinal flora aggravate lung inflammation and lung tissue damage by generating harmful metabolic products. SCFAs in the intestinal tract are mainly produced by the breakdown of dietary fiber by intestinal flora. Exposure to tobacco particles reduces SCFA levels in the feces of COPD mice. After administration of oral antibiotics to clear SCFAs from the intestines, emphysema and lung inflammation were exacerbated, demonstrating that SCFAs are involved in the inflammatory response and emphysema in COPD. Subsequently, in a mouse model of lung emphysema established using trypsin, the levels of SCFAs did not decrease. This finding indicates that the decrease in intestinal SCFA levels was not related to simple lung emphysema but was related to systemic inflammatory responses (84). This suggests that SCFAs a therapeutic approach for COPD hold significant potential.

## 5 Treatment

The heterogeneity of COPD may depend on the pulmonary microbiome and immune status of the host. In a clinical study that grouped patients with AECOPD based on differences in microbiota and airway inflammatory mediator levels, the abundance of Proteobacteria and the Proteobacteria/Firmicutes ratio in the pulmonary flora were higher in patients with elevated neutrophil counts and increased levels of inflammatory factors IL-1β, IL-6, and TNF-α. Similarly, in patients with eosinophilic infiltration and higher levels of the Th2 inflammatory mediators IL-5, IL-33, chemokine (C–C motif) ligand 13 (CCL13), and chemokine (C–C motif) ligand 17 (CCL17), α-diversity of the lung microbiota was high, with Bacteroidetes as the main flora. These patients also exhibited increased levels of type 1 inflammatory mediators such as chemokine ligand (CXCL)10, CXCL11, and IFN-γ, along with higher proportions of pulmonary bacteria, including Actinobacteria, Firmicutes, and *Streptococcus*. These findings provide a theoretical basis for personalized treatment of COPD based on the microbiome. The following outlines the treatment strategies (**Table 2**).

**Table 2:** Therapeutic strategies targeting the gut–lung axis in PM-induced COPD.

Therapeutic approach	Supporting evidence	Outcomes	Underlying mechanisms	References
Microbiota-targeted interventions				
*Bifidobacteria* and *Lactobacilli*	Clinical	(1) Improves pulmonary function. (2) Relieves clinical symptoms.	Alters the gut microbiota: *Lactobacillus* and *Bifidobacterium* increased, while *Enterobacteriaceae* and *Enterococcus* decreased.	(104)
*Lactobacillus plantarum* KC3 (KC3)	Animal	(1) Reduces lung inflammation: TNF-α decreased. (2) Reduces the infiltration of immune cells in the lungs: neutrophils decreased.	(1) Alters the gut microbiota. (2) Regulates colonic immunity: Treg cells increased.	(109)
Fecal microbiota transplantation	Animal	(1) Alleviates emphysema. (2) Reduces lung inflammation: IL-1β, IL-8, IL-6, and IFN-γ decreased.	(1) Balances microbiota in the intestines: the Firmicutes/Bacteroides (F/B) ratio was reduced. (2) Alters metabolism in the intestine: the concentration of SCFAs increased.	(112)
Dietary factors				
High-fiber diet	Animal	(1) Reduces the infiltration of immune cells in the lungs: lymphocytes and macrophages decreased. (2) Reduces lung inflammation: IL-6 and IFN-γ decreased. (3) Improves lung pathological damage: emphysema was alleviated.	(1) Balances microbiota in the intestines: the Firmicutes/Bacteroides (F/B) ratio was reduced. (2) Alters metabolism in the intestine: the concentration of SCFAs increased.	(112)
Supplement butyrate	Animal	(1) Reduces lung inflammation. (2) Regulates pulmonary immunity.	Regulates gut immunity: the proportions of IL-2 decreased.	(98)
Immunomodulator				
OM-85	Animal	(1) Partial recovery of pulmonary function. (2) Improves lung pathological damage. (3) Reduces lung inflammation: TNF-α and IL-17 decreased.	(1) Microbiota balance in the intestines: Firmicutes and Proteobacteria increased. (2) Balances the microbiota in the lungs: microbial diversity increased.	(117)
Antibiotics				
Macrolide antibiotics	Clinical and animal	Reduces airway inflammation in mice: IL-8 and IL-6 levels in BALF decreased.	(1) Alters the lung microbiota in COPD patients: the abundance of airway microbiota decreased, without changes in diversity. (2) Regulates the intestinal microbiota in COPD patients: Firmicutes increased, while Proteobacteria and Bacteroidetes decreased. (3) Regulates the lung microbiota and metabolism in mice. (4) Regulates the intestinal microbiota in mice.	(118)
Traditional Chinese medicine				
Seabuckthorn Wuwei Pulvis	Animal	(1) Improves pulmonary function: FEV0.3, FVC, and FEV0.3/FVC increased. (2) Reduces lung inflammation: TNF-α, IL-8, IL-6, and IL-17 decreased.	(1) Reduces intestinal permeability: enhances intestinal tight junction protein expression (ZO-1, occludin-1). (2) Balances microbiota: increases beneficial SCFA-producing bacteria in the intestines.	(124)
Qifenggubiao granules	Animal	(1) Improves pulmonary function: FEV20/FVC increased, while FRC decreased. (2) Reduces lung inflammation: TNF-α and IL-1β decreased, while IL-10 increased. (3) Reduces lung damage.	(1) Microbiota balance in the intestines: decreases the p_Firmicutes/p_Bacteroidetes ratio. (2) Regulates intestinal immunity: restores the polarization balance of M1 and M2 in the intestines.	(123)
Qibai Pingfei Capsule	Animal	Improves pulmonary function: FEV0.3 and FEV0.3/FVC increased.	(1) Regulates the intestinal microbiota: Coprococcus_2 and Blautia decreased, while Prevotellaceae_UCG_003 increased. (2) Regulates the lung microbiota: mycoplasma decreased, while Rikenellaceae increased. (3) Regulates pulmonary immunity: Th17 decreased, while Treg increased.	(122)

### 5.1 Microbiota-targeted interventions

Supplementation with probiotics can protect the respiratory system by maintaining the stability of intestinal flora, preserving epithelial barrier integrity, and supporting antioxidant and immunoregulatory functions. A single-center clinical study found that budesonide combined with *Bifidobacterium* and *Lactobacillus* effectively restored the balance of intestinal microbes and improved lung function by increasing the abundance of the beneficial gut bacteria *Lactobacillus* and *Bifidobacterium*, while decreasing the abundance of *Enterobacteriaceae* and *Enterococcus* (104). Probiotics can increase the Firmicutes/Bacteroidetes ratio and the abundance of beneficial bacteria in the intestine while reducing the abundance of proinflammatory bacteria, thereby mitigating alterations in intestinal flora caused by PM_2.5_ stimulation (105). *Lactobacillus* and *Bifidobacterium* are important probiotics in the gut, and oral administration of *Lactococcus* and *Bifidobacteria* can reduce the abundance of Proteobacteria, which produce proinflammatory factors such as TNF-α and IL-6 (106). Therefore, personalized intervention programs can be developed based on the microbial characteristics of patients with COPD.

*In vitro* studies have shown that *Lactobacillus* promotes the proliferation and migration of intestinal epithelial cells, regulates the expression of tight junction proteins claudin-1 and occludin, reduces paracellular permeability, and prevents the nuclear translocation of NF-κB p65 in intestinal epithelial cells, thereby inhibiting the release of proinflammatory cytokines TNF-α and IL-1β (107, 108). In the lungs of mice, *Lactobacillus plantarum* inhibits the recruitment of neutrophils and the production of proinflammatory cytokines IL-17 and TNF-α induced by air pollutants, exerting a protective effect on the respiratory system (109). By reducing the production of cellular ROS, *Lactobacillus* exerts an antioxidant effect, mitigating lung tissue damage caused by oxidative stress (110). In addition, probiotics demonstrate immunoregulatory activities by modulating the Th17/Treg balance to inhibit the release of the proinflammatory cytokine IL-17A, which recruits neutrophils via chemotaxis; enhance the release of anti-inflammatory factors, including transforming growth factor (TGF)-β and IL-10; and reduce the lung inflammatory damage induced by PM_2.5_ (80, 105).

FMT may represent another novel therapeutic approach for COPD (111). In CS-induced emphysema animal models, FMT from healthy donors significantly ameliorated emphysema and reduced levels of IL-6, γ-IFN, IL-1β, and TNF-α in lung tissue. More importantly, the gut microbiota in FMT-treated animals was restored toward a healthy state and produced higher levels of SCFAs, which were associated with improvements in alveolar destruction (112).

### 5.2 Dietary factors

Dietary modification strategies can improve COPD through the gut–lung axis, with SCFAs playing a key protective role. Daily intake of dietary fiber increases intestinal SCFA levels, alleviates emphysema, and reduces the release of the proinflammatory cytokines such as IL-6 and IFN-γ, thereby mitigating the symptoms of COPD (112). Dietary fiber intake not only affects the Firmicutes/Bacteroidetes ratio, altering the composition of intestinal and pulmonary microbiota, but also promotes the formation of macrophages and dendritic cell (DC) precursors, thereby modulating the pulmonary immune microenvironment (113). *Clostridium* in the intestine is also a source of SCFAs. In patients with AECOPD, the abundance of *Clostridium intestinalis* decreases, reducing butyrate production and thereby limiting its inhibitory effect on intestinal ILC2 activation to iILC2. Supplementation with butyrate can inhibit the activation and proliferation of intestinal innate lymphoid cells, reduce the release of proinflammatory cytokines, and attenuate inflammation in lung tissues (98).

### 5.3 Immunomodulators

Oral bacteria-derived immunomodulatory drugs and thymosin drugs can regulate respiratory immunity and reduce the risk of AECOPD by immunizing lymphocytes through the intestinal mucosa. Oral bacteria-derived immunomodulatory drugs include OM-85, Megloniella albus, Bistoid, and Ismigen. These drugs have similar mechanisms: after oral administration, they act as PAMPs and are recognized by pattern recognition receptors (PRRs) and TLRs on innate immune cells in intestinal mucosa-associated lymphoid tissue, inducing DC maturation and activation. This stimulates DCs to produce chemokines and B-cell-activating factor (BAFF) and activates natural killer cells and macrophages within the intestinal mucosa-associated lymphoid tissue. They induce the differentiation of naïve T lymphocytes into Th1 and Th17 cells, promoting the production of proinflammatory cytokines and chemokines, and stimulate the differentiation of Treg cells to maintain immune balance. Moreover, they inhibit Th2-type allergic reactions and promote the differentiation of B lymphocytes into immunoglobulin A (IgA). These activated immune cells, cytokines, and immunoglobulins enter the lung tissue via lymphatic homing and the circulatory system. Ultimately, they enhance respiratory mucosal immunity and exert anti-inflammatory effects (114–117).

### 5.4 Antibiotics

Macrolide antibiotics can regulate immunity and alter the composition of both lung and intestinal microbiota. A recent study showed that long-term erythromycin treatment could modulate lung and intestinal flora and correct metabolite disturbances in mice and patients with COPD, reduce the abundance of pathogenic bacteria, and increase the number of symbiotic bacteria (118). Azithromycin significantly reduces the frequency of acute exacerbations in patients with COPD (119). It not only interacts with tight junction proteins in epithelial cells to maintain the integrity of the physical barrier but also regulates DCs, neutrophils, macrophages, and the differentiation of T lymphocyte subgroups, thereby inhibiting the release of proinflammatory cytokines and chemokines to achieve immune regulation (120). However, azithromycin can alter the stability of the airway microbiota in healthy adults, reducing the airway’s resistance to outdoor PM_2.5_, indicating the importance of rational antibiotic use (121).

### 5.5 Traditional Chinese medicine

Multiple studies have indicated that traditional Chinese medicine (TCM) treatment can slow the progression of COPD (122, 123). Its mechanism is not limited to direct anti-inflammatory effects in the lungs, but also includes its ability to reduce intestinal permeability and decrease endotoxin translocation. By doing so, it cuts off the transmission of gut-derived inflammation to the lungs, reduces inflammatory cell infiltration in lung tissue, and lowers levels of IL-17, TNF-α, IL-8, and IL-6. These effects ultimately improve lung function and slow COPD progression (124).

TCM can also modulate immune responses in both the lungs and the gut. Qifeng Gubiao Granules not only correct the imbalance of M1/M2 macrophages in the lungs and spleen of COPD mice, but, more importantly, they significantly regulate M1 and M2 macrophages in the intestine. This finding indicates that Qifeng Gubiao Granules directly target intestinal immunity, subsequently transmit immunoregulatory signals to the lungs, and ultimately drive pulmonary macrophages to polarize toward the anti-inflammatory M2 phenotype. These results provide direct experimental evidence that TCM can ameliorate immune imbalance via the gut–lung axis (123). Furthermore, Qibai Pingfei Capsule has been shown to maintain the Th17/Treg immune balance in the lungs of COPD rats and improve pulmonary immune status. This effect is associated with alterations in both the lung and gut microbiota (122).

TCM regulates the gut microbiota of COPD mice. Specifically, since a decrease in the Firmicutes/Bacteroidetes (F/B) ratio in the gut is associated with reduced inflammation, Qifeng Gubiao Granules have been shown to decrease the abundance of p:Firmicutes and increase p:Bacteroidetes in the gut of COPD mice, thereby reducing inflammation (123). They also promote the production of beneficial metabolites, such as SCFAs, by the gut microbiota (124). These substances not only supply energy to the intestinal mucosa but also travel via the bloodstream to regulate lung immune cell function, thereby reducing airway inflammation and immune dysregulation in COPD. Furthermore, Qibai Pingfei Capsule exerts protective effects against COPD by mediating changes in the lung microbiota via the gut–lung axis (122).

## 6 Conclusions and prospects

PM affects the progression of COPD through the gut–lung axis, indicating a shift in understanding COPD from a focus on local lung tissue to a systemic perspective. This review emphasizes that immune cells, intestinal microbiota, and metabolites are central to the interaction within the gut–lung axis and elaborates on the mechanisms by which the gut–lung axis contributes to PM-induced COPD.

At the macroscopic level, epidemiological evidence has shown that exposure to PM increases the incidence, acute exacerbation rate, and mortality rate of COPD (14–17) and is also associated with a higher incidence of gastrointestinal diseases (71). Patients with COPD have an increased risk of developing inflammatory bowel disease (125), and conversely, inflammatory bowel disease increases the risk of death from COPD (126). This connection between the lungs and the intestines suggests a shared underlying link.

At the microlevel, PM exposure not only leads to the infiltration and activation of immune cells, such as macrophages, neutrophils, and lymphocytes, in the lungs and intestines (28, 72) but also causes significant disruptions in microbial communities in distant organs through immune cell homing and the leakage of immune mediators. More importantly, in the circulatory system (11), levels of inflammatory factors released into the blood and microbial metabolites increase simultaneously, providing direct evidence for bidirectional communication between the gut and the lungs.

The core of the interaction within the gut–lung axis lies in self-circulation. First, from the lungs to the intestines, PM entering the lungs activates the pulmonary immune system, releasing immune mediators such as TNF-α and IL-6 into the bloodstream. These mediators indirectly damage the intestines by disrupting the balance of intestinal microorganisms and impairing epithelial barrier function. Moreover, pathogenic bacteria in the lungs of COPD patients increase due to PM exposure; these bacteria can migrate to the intestines and cause further damage. Second, after PM reaches the intestine through swallowing or the circulatory system, it disrupts the intestinal flora and damages the intestinal epithelial barrier, increasing intestinal permeability. This, in turn, allows proinflammatory mediators to translocate into the circulatory system, triggering a systemic low-level inflammatory state. Undoubtedly, this exacerbates the inflammatory load in the lungs of COPD patients.

Based on the above mechanisms, targeting the gut–lung axis offers a new perspective for the treatment of COPD. Combining systemic therapies aimed at restoring homeostasis within the gut–lung axis with traditional anti-inflammatory and bronchodilator drugs provides a novel, comprehensive management strategy for COPD progression caused by PM exposure. However, the most fundamental approach remains the reduction of sources and exposure to environmental pollutants.

However, the effects of PM on the gut–lung axis in COPD are still at an early stage of investigation. First, as PM is a complex mixture, future studies should identify which specific components cause detailed changes in microorganisms. Second, longitudinal studies are needed to determine how protective mechanisms are activated in the early stages of long-term air pollution exposure and when the decompensation of these protective mechanisms begins. Moreover, although some studies have clarified the molecular mechanisms by which microbial metabolites, such as SCFAs and trimethylamine *N*-oxide (TMAO), act on cells, the molecular mechanisms by which PM contributes to COPD through microorganisms and immunity remain to be further explored. It is anticipated that the gut–lung axis will become a novel therapeutic target for COPD associated with PM exposure.
